# Reconstructed Metabolic Network Models Predict Flux-Level Metabolic Reprogramming in Glioblastoma

**DOI:** 10.3389/fnins.2016.00156

**Published:** 2016-04-18

**Authors:** Emrah Özcan, Tunahan Çakır

**Affiliations:** Computational Systems Biology Group, Department of Bioengineering, Gebze Technical UniversityGebze, Turkey

**Keywords:** aerobic glycolysis, glutaminolysis, constraint-based models, omics data, tumor subtypes, GBM-specific metabolic model

## Abstract

Developments in genome scale metabolic modeling techniques and omics technologies have enabled the reconstruction of context-specific metabolic models. In this study, glioblastoma multiforme (GBM), one of the most common and aggressive malignant brain tumors, is investigated by mapping GBM gene expression data on the growth-implemented brain specific genome-scale metabolic network, and GBM-specific models are generated. The models are used to calculate metabolic flux distributions in the tumor cells. Metabolic phenotypes predicted by the GBM-specific metabolic models reconstructed in this work reflect the general metabolic reprogramming of GBM, reported both in *in-vitro* and *in-vivo* experiments. The computed flux profiles quantitatively predict that major sources of the acetyl-CoA and oxaloacetic acid pool used in TCA cycle are pyruvate dehydrogenase from glycolysis and anaplerotic flux from glutaminolysis, respectively. Also, our results, in accordance with recent studies, predict a contribution of oxidative phosphorylation to ATP pool via a slightly active TCA cycle in addition to the major contributor aerobic glycolysis. We verified our results by using different computational methods that incorporate transcriptome data with genome-scale models and by using different transcriptome datasets. Correct predictions of flux distributions in glycolysis, glutaminolysis, TCA cycle and lipid precursor metabolism validate the reconstructed models for further use in future to simulate more specific metabolic patterns for GBM.

## Introduction

Among malignant brain tumors, the most common one is glioblastoma (glioblastoma multiforme, GBM). It is also one of the most lethal cancer types, with a 5-year survival rate of only 3%, compared to an average of 30% for other types of brain tumors (Ostrom et al., 2013). This demands for a well-characterization of molecular mechanisms of glioblastoma cells to develop treatment strategies. Therefore, it is crucial to build computer models which can mimic major characteristics of the cancerous cells (Folger et al., 2011; Hadi and Marashi, 2014; Ghaffari et al., 2015; Yizhak et al., 2015). In glioblastoma, the most significant reprogramming occurs in the metabolic machinery of the cells. Major alterations associated with cancer metabolism such as Warburg effect (Shlomi et al., 2011) are also observed in glioblastoma (DeBerardinis et al., 2007; Wolf et al., 2010). Major ATP source is via aerobic glycolysis, although TCA cycle is still slightly active according to recent reports (DeBerardinis et al., 2007; Wolf et al., 2010; Ru et al., 2013). Another characteristics of metabolic remodeling of glioblastoma is the uptake of glutamine, which contributes to the replenishment of TCA cycle intermediates (DeBerardinis et al., 2007). Several other metabolic alterations occur in the metabolic flux patterns in glioblastoma, mostly due to increased flux toward lipid and nucleotide synthesis to sustain growth.

The systems approach to biology and medicine led to a number of computational approaches to study network-based alterations in cells in response to perturbations. The study of cancer metabolism via computational approaches has therefore shown a sharp increase in the last decade (Ghaffari et al., 2015; Yizhak et al., 2015). Genome-scale metabolic modeling is one of the highly preferred computational methods since it allows the investigation of the cellular flux state in genome-scale by only incorporating few constraints (Kim et al., 2012; Mardinoglu et al., 2013; Bordbar et al., 2014). A generic genome-scale metabolic model includes all potential biochemical reactions to be used by the associated organism. There are computational methods which process generic metabolic models by integrating with omics data such that condition specific metabolic models are reconstructed (Blazier and Papin, 2012; Saha et al., 2014). Such methods can be divided into two groups in terms of their algorithmic approach. The first group uses context-specific omics data directly to improve the prediction of metabolic flux distributions, such as E-Flux (Colijn et al., 2009), PROM (Chandrasekaran and Price, 2010), MADE (Jensen and Papin, 2011), tFBA (van Berlo et al., 2011), TEAM (Collins et al., 2012), and RELATCH (Kim and Reed, 2012). The second group processes the data to create context-specific models from generic metabolic models, such as GIMME (Becker and Palsson, 2008), iMAT (Shlomi et al., 2008), INIT (Agren et al., 2012), AdaM (Töpfer et al., 2012), mCADRE (Wang et al., 2012), and EXAMO (Rossell et al., 2013), which can later be used for flux calculation. It was shown that the two approaches have no clear superiority over each other (Machado and Herrgård, 2014).

There are studies which integrate omics data with the metabolic models in order to reconstruct context-specific cancer metabolic models. Human metabolic reconstruction Recon1 (Duarte et al., 2007) was used to reconstruct the first generic genome-scale model of cancer, aiming to capture main metabolic functions of many cancer types using cancer gene expression data (Folger et al., 2011). Agren et al. reconstructed a generic genome-scale human metabolic model, to create genome-scale active metabolic networks for 69 different cell types including 16 cancer types using tissue specific proteome data (Agren et al., 2012). Recently published reviews (Ghaffari et al., 2015; Yizhak et al., 2015) survey the studies of cancer metabolism by reconstructed metabolic model approaches and discusses the challenges such approaches face.

In this study, metabolic alteration of glioblastoma was investigated using *in-silico* metabolic model reconstruction approach. The genome-scale brain metabolic model (Sertbas et al., 2014) reconstructed recently by our group was first modified by adding biomass growth reaction to reflect the tumor proliferation. Afterwards, the glioblastoma gene expression data from Gene Omnibus Database (Edgar et al., 2002) were integrated with the growth-implemented brain specific metabolic model to obtain GBM-specific metabolic models. The models predict major flux-level metabolic alterations and reprogramming associated with GBM, giving consistent results with both *in-vitro* and *in-vivo* studies.

## Materials and methods

### Genome-scale brain metabolic network for brain tumors

The genome-scale brain metabolic model *iMS570* (Sertbas et al., 2014; Cakir, in press) reconstructed previously by our group possesses 630 metabolic reactions in and between astrocyte and neurons, which are controlled by 570 genes. *iMS570* includes the fundamental pathways such as central carbon metabolism (glycolysis, pentose phosphate pathway, TCA cycle), lipid metabolism, nucleotide metabolism, amino acid metabolism (synthesis and catabolism), the well-known glutamate-glutamine cycle, other coupling reactions between astrocytes and neurons, and neurotransmitter metabolism. In total, 42 pathways are covered by the model. *iMS570* does not have a growth reaction to simulate the proliferation of brain tumors since mammalian brain cells do not grow in non-tumor states. Therefore, an extended literature survey was performed to define a growth reaction for tumor proliferation in brain (See Supplementary File 1). The modified model which can grow *in-silico* thanks to the included biomass growth reaction is called *iMS570*^*g*^. The biomass composition was defined based on brain white matter since GBM is mostly observed in this tract as the parent tissue (Bohman et al., 2010; Omuro and DeAngelis, 2013; Cuddapah et al., 2014). Brain white matter has a high composition of lipid (54.9%) and protein (39.5%) (Brady et al., 2012). The percentages of glial and neuronal cells in the white matter were reported to be 94 and 6% respectively in a recent study by using a novel method based on tagging the DNA inside the nuclei with fluorescent proteins (Azevedo et al., 2009). These values were used to define the relative contributions of astrocyte and neuron cells to the biomass reaction in the model. Free amino acid composition for human brain reported by (Banay-Schwartz et al., 1992, 1993a,b) was used as the amino acid composition of the protein pool in the biomass reaction. (See Supplementary File 1 for details on biomass composition and contribution of cell types). One characteristic of GBM cells is the altered glutamine metabolism which manifests itself as decreased glutamine production, high glutamine uptake rate and glutaminolysis (Portais et al., 1993; DeBerardinis et al., 2007). Four extra reactions denoting the glutamine uptake and glutaminolysis metabolism were also included in *iMS570*^*g*^ in order to cover the tumor-caused alterations in glutamine metabolism (See Supplementary File 1).

### GBM transcriptome datasets

Lee et al. used several published GBM transcriptome datasets in addition to their own study to investigate survival differences between GBM subtypes (Lee et al., 2008). The whole transcriptome dataset is stored in the public transcriptome database, GEO (Edgar et al., 2002), under GSE13041. The dataset covers gene expression data from different microarray platforms. We focused on a subset of the data from two different platforms (GPL96, Affymetrix Human Genome U133A Array and GPL570, Affymetrix Human Genome U133 Plus 2.0 Array) and analyzed them separately to document the effect of platform type on the results. A previous study defined three distinct subtypes of GBM tumors based on clustering analysis of transcriptome data (Phillips et al., 2006): Mesenchymal (Mes), ProNeural (PN), Proliferative (Pro). Here, PN type has a better prognosis, and has a more similar gene expression profile to normal brain and neurogenesis. The other two types have poor prognosis, and show resemblance to proliferative or mesenchymal-origin cells in terms of gene expression (Phillips et al., 2006). The data from GPL96 platform was analyzed by considering this classification, which was already implemented by the authors (Lee et al., 2008). Another dataset by Mangiola et al. (2013) was also used in this study. They investigated the relation between peritumoral tissue (brain adjacent to tumor) and GBM using gene expression profiles. Normal white matter was used as a control group. The transcriptome dataset is based on GPL96 platform, and it is stored in GEO database under GSE13276. The reason behind using another dataset was to test the effect of different datasets on the bioinformatic algorithms used in this study.

In total, five different GBM transcriptome datasets were formed for the purpose of this study: Three datasets of GBM subtypes for GPL96 platform of GSE13041, a dataset of GPL570 platform for GSE13041, and a dataset from GSE13276. All GBM samples used in our study were collected from tumor biopsies of GBM patients.

### Obtaining GBM-specific metabolic models

**Figure 1:**
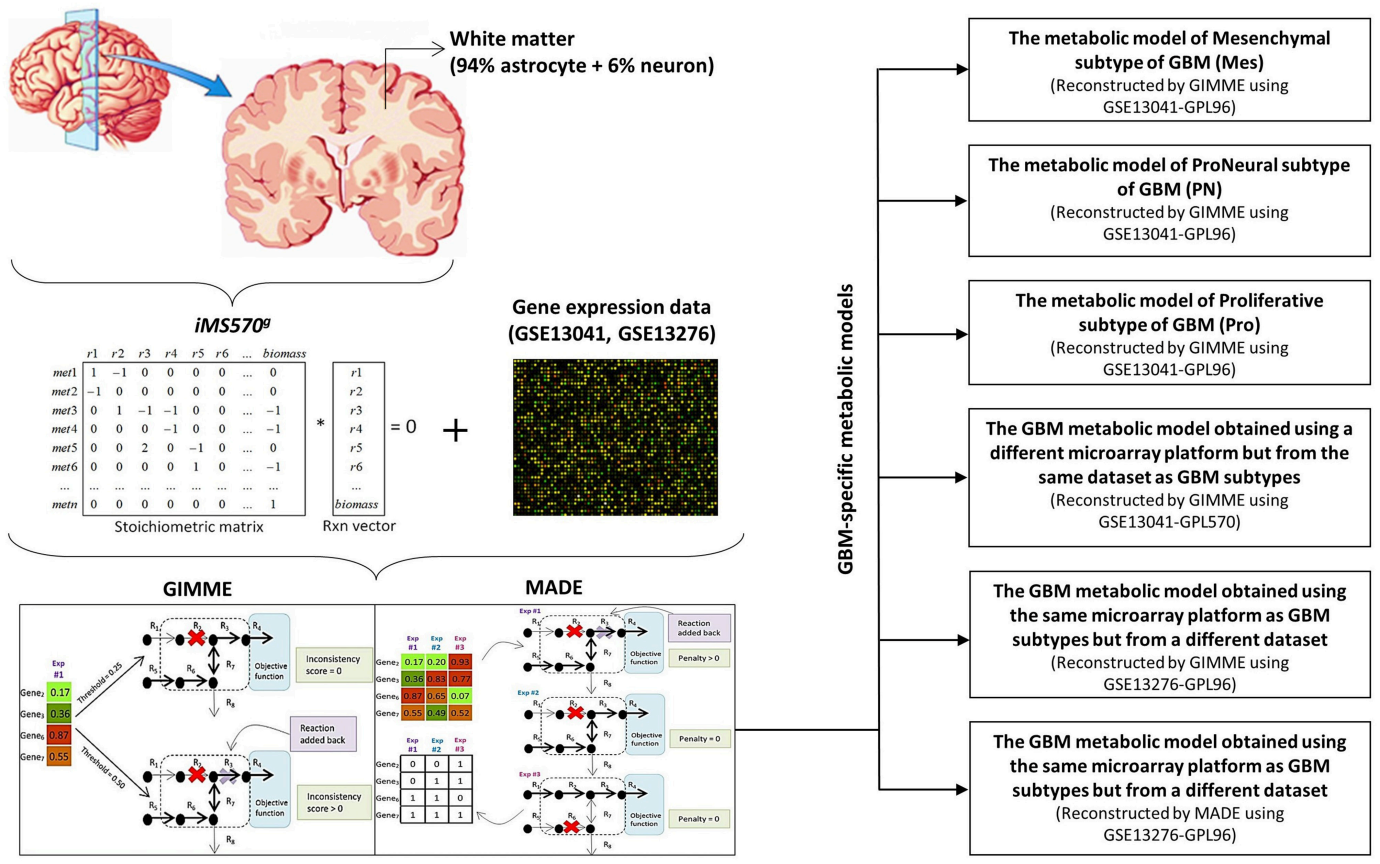
**Recontruction of the GBM metabolic models**. GBM gene expression data were integrated with the growth-implemented brain specific genome-scale metabolic model (*iMS570*^*g*^) by GIMME and MADE algorithms to create GBM metabolic models. The algorithms are shown in paranthesis for related GBM metabolic models. (Mes, Mesenchymal subtype of GBM; PN, ProNeural subtype of GBM; Pro, Proliferative subtype of GBM). GIMME and MADE sketches were obtained from Figure 1 of Blazier and Papin (2012).

*iMS570*^*g*^, the growth-implemented brain specific genome-scale metabolic network, was integrated with the GBM gene expression data mentioned in the previous section to generate context-specific GBM metabolic models and metabolic flux distributions. Two alternative methods, GIMME (Becker and Palsson, 2008) and MADE (Jensen and Papin, 2011), were applied to generate GBM metabolic models and test the effect of different algorithms on the results (Figure 1). Friedmann-Morvinski et al. (2012) showed that GBM can originate not only from astrocytes but also from neurons. Therefore, GBM transcriptome data were mapped to both astrocytic and neuronal reactions in *iMS570*^*g*^ in order to generate GBM metabolic models via GIMME and MADE. While the output of MADE is a context-specific flux distribution, the output of GIMME is a context-specific model which needs to be further processed to obtain a flux distribution.

#### GIMME

GIMME (Gene Inactivity Moderated by Metabolism and Expression) algorithm uses binarized gene expression data and a genome scale metabolic network to generate a context-specific reconstruction such that the highest consistency with the available data is ensured (Becker and Palsson, 2008). All five transcriptome datasets were used as experimental soft-constraints to obtain corresponding GBM-specific metabolic models using GIMME algorithm. Transcriptome data were first binarized based on a specified threshold to obtain highly and lowly expressed genes. GIMME algorithm removes the reactions which correspond to gene expression levels below the specified threshold, and the algorithm adds a removed reaction back if the metabolic model cannot achieve the desired functionality. The desired functionality was used as biomass growth reaction in *iMS570*^*g*^. The threshold criteria for GIMME in this study was that the threshold must not be higher than the levels of some genes known to be upregulated in GBM. These genes are HK2, PKM2, GLS, ACLY, ACC, and FASN, which take roles in glucose, glutamine or lipid metabolisms (Wolf et al., 2010; Ru et al., 2013). Considering this criteria, we chose 1/2, 1/1, and 1/3 of “the mean of transcriptome data of related GBM dataset” as the thresholds in GIMME algorithm for GSE13041 (GPL96), GSE13041 (GPL570), and GSE13276 respectively. Five percent sensitivity analysis was applied for the chosen thresholds, and no significant change was observed in the calculated flux distributions (data not shown). The genes whose expression levels are higher than the threshold were assumed highly expressed and set to “1,” and the genes whose expression levels are lower than the threshold were assumed lowly expressed and set to “0,” to be used as an input to GIMME. GIMME functionality of COBRA (COnstraint-Based Reconstruction and Analysis; Schellenberger et al., 2011) Toolbox was used under MATLAB (MathworksInc., Natick, MA, USA) environment to run the algorithm. The number of the removed reactions by GIMME from *iMS570*^*g*^ was 57, 55, and 54 for Mes, PN and Pro subtypes. For GPL570 based data 48 reactions were removed whereas the number was 34 for GSE13276 dataset. Finally, five different GBM-specific metabolic models were reconstructed by GIMME. Then, flux balance analysis (FBA) (Orth et al., 2010) for maximizing biomass growth rate as primary objective function with subsequent minimization of Euclidean norm of internal fluxes was applied to five different GBM models obtained by GIMME algorithm. This dual objective function framework was shown to give better results (Cakir et al., 2007; Tarlak et al., 2014) since it ensures minimal use of enzyme resources to achieve the primary objective. GBM models corresponding to the five transcriptome datasets and the growth-implemented model are available in SBML format in Supplementary File 2.

#### MADE

In addition to GIMME, we used MADE algorithm to see if there is any difference between the methods that maps transcriptome data on metabolic models to obtain condition-specific models. MADE (Metabolic Adjustment by Differential Expression) algorithm uses the expression levels of significantly changed genes or proteins to generate a functional metabolic model that most accurately recapitulates the expression dynamics (Jensen and Papin, 2011). MADE eliminates the probable problems of arbitrary user-specified threshold, as employed by GIMME, by using statistically significant changes in gene expression measurements between two conditions to determine highly and lowly expressed genes (Blazier and Papin, 2012). Since MADE requires a control group for the analysis, only transcriptome data GSE13276 was used in the MADE-based analysis. MADE algorithm requires three inputs to generate a context-specific flux distribution, which are a genome-scale metabolic model, fold changes and *p*-values of gene expression levels between the compared conditions (Jensen and Papin, 2011). Fold changes and *p*-values calculated by student's *t*-test were based on the white matter data as a control group. The objective function used by MADE algorithm for the *iMS570*^*g*^ was biomass growth reaction, as used in GIMME. MADE algorithm was used via TIGER (Toolbox for Integrating Genome-scale Metabolism, Expression, and Regulation; Jensen et al., 2011) toolbox under MATLAB environment. MADE uses its own flux calculation algorithm which is based on mixed integer linear programming. Both GIMME and MADE were run in default settings, and GUROBI optimizer (http://www.gurobi.com) was used as a solver in both tools.

### Constraints reflecting physiology of glioblastoma multiforme (GBM)

GIMME was run with the constraints which reflect basic characteristics of GBM. The reactions defining glutamine exchange from astrocyte to neuron (r_95_) and glutamine release (r_580_) were constrained as zero due to the fact that glutamine exchange between astrocytes and neurons in healthy brain is perturbed within GBM (Marin-Valencia et al., 2012). Glycogen uptake (r_575_) and ketone body metabolism (r_608_, r_609_), which are used as alternative pathways in case of low activity of the glucose metabolism (Cakir et al., 2007), were also constrained to zero in our study. NH_3_ exchange reaction (r_607_), which is defined only as uptake reaction in *iMS570*, were changed to a reversible exchange reaction to allow NH_3_ release as observed in the GBM (DeBerardinis et al., 2007). Also a ratio, not an absolute value, of 94/6 was defined as a constraint for relative glucose, glutamine, and oxygen uptake rates of astrocytes and neurons considering the relative amounts of the two cell types in brain white matter (see below). GIMME generated GBM specific metabolic networks based on these constraints. Afterwards, corresponding flux distributions were calculated by employing more specific additional constraints as follows: Glucose, glutamine and oxygen uptake rates were fixed to the experimental flux values indicated in the study of DeBerardinis et al. (2007), which are 0.852 mmol/gDW/h, 0.080 mmol/gDW/h, and 0.272 mmol/gDW/h respectively. These uptake rates were distributed among astrocyte and neuron as 94 and 6% respectively, according to the relative amount of the cell types in white matter (Azevedo et al., 2009). Furthermore, the upper bound of the uptake rates of the amino acids other than glutamine were constrained to one tenth of the glutamine uptake rate because Yang et al. (2009) observed that GBM cells consume glutamine at a rate at least 10-fold higher than any other amino acids. Complete list of the constraints used in the models are also given in Supplementary File 1. All the above mentioned constraints were directly used as input to MADE algorithm since, in contrast to GIMME, it calculates flux distribution as a direct output. Although the contribution of neuronal reaction rates is usually considerably lower, this contribution was accounted by summing up astrocytic fluxes with neuronal counterparts to evaluate the phenotype of GBM metabolic models in the results part.

## Results

The GBM metabolic models used in this study were reconstructed by integrating GBM gene expression data with the generic genome-scale brain metabolic model, *iMS570*^*g*^ (See Materials and Methods). Five GBM metabolic models were created by GIMME, three for the comparison of Mes, PN, and Pro subtypes of GBM, one to compare the effect of different microarray platforms (The GBM metabolic model obtained using a different microarray platform but from the same dataset as GBM subtypes), one to compare the effect of different datasets (The GBM metabolic model obtained using the same microarray platform as GBM subtypes but from a different dataset). *In-silico* GBM phenotypes obtained via metabolic modeling were compared with the literature based experimental results. Metabolic fluxes calculated for healthy brain in resting state (Sertbas et al., 2014) were also used for comparison.

### GBM subtypes

**Figure 2:**
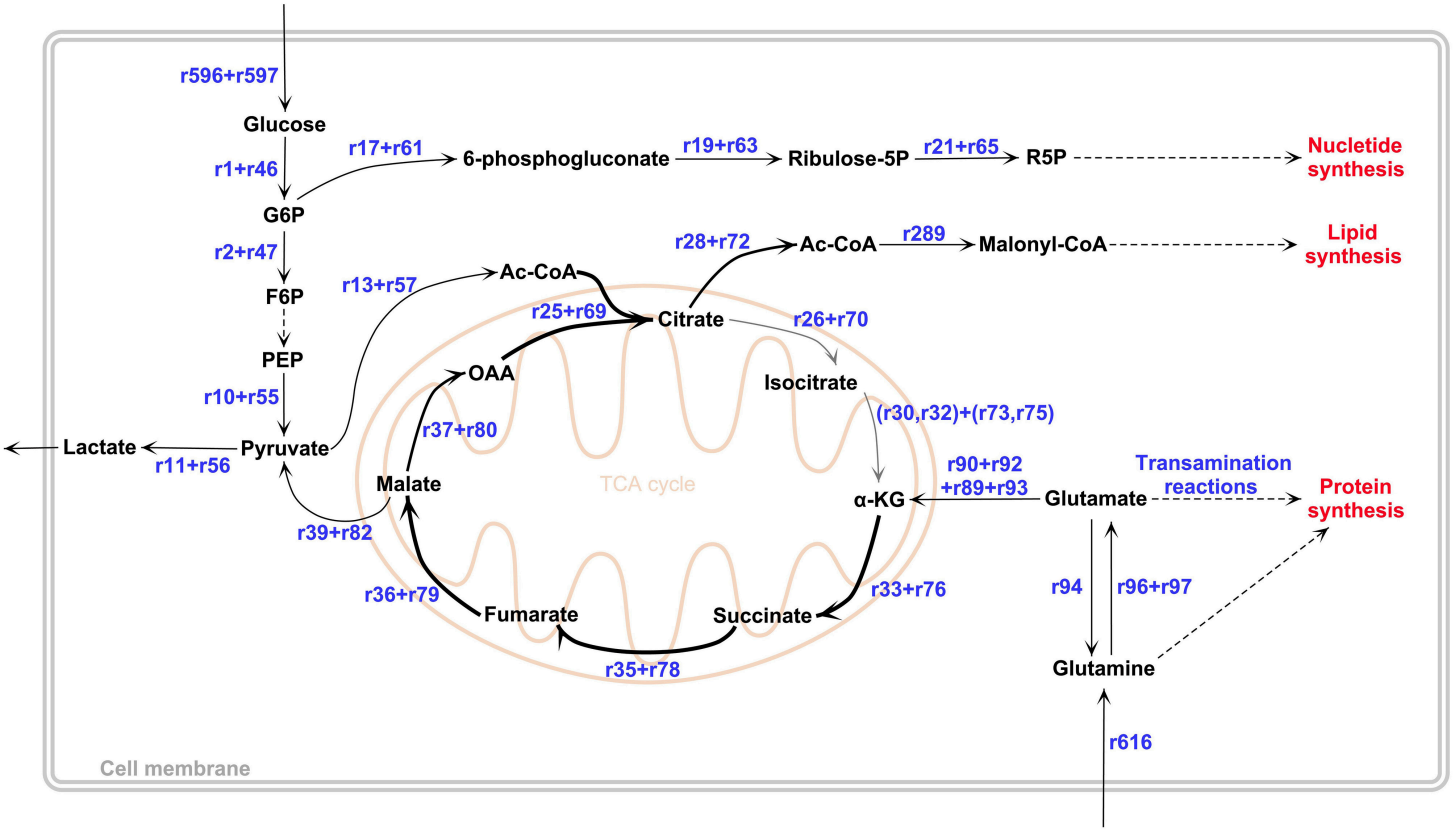
**GBM metabolic remodeling reported in literature**. In TCA cycle, low-flux reactions were represented by a thinner gray arrow. Our computational results obtained by all GBM metabolic models support this remodeling topology. All reaction IDs, shown also in the figure, and corresponding reactions can be found in Supplementary File 1. The figure was drawn in PathVisio 3 toolbox (Kutmon et al., 2015).

The GBM subtypes, Mesenchymal (Mes), ProNeural (PN), and Proliferative (Pro) GBM, are classified based on the clustering of gene expression profiling (Phillips et al., 2006; Lee et al., 2008; Verhaak et al., 2010; Huse et al., 2013). *In-silico* GBM subtype metabolic models for Mes, PN and Pro types were reconstructed by GIMME algorithm (Becker and Palsson, 2008; see Materials and Methods) and used in the calculation of metabolic fluxes. Key fluxes and flux ratios are presented in Table 1. The results in Table 1 show high qualitative and quantitative agreements between the flux predictions and the literature results. Results reveal subtle differences between the GBM subtypes in terms of simulated metabolic flux phenotypes. However, all GBM subtype metabolic models exhibit the same behavior in terms of active flux routes. This flux routing, as shared by the three subtypes, are summarized in Figure 2. The simulation results depicted in the figure are in agreement with the major properties of GBM metabolic phenotypes reported in literature. The results confirm the study which reports similar metabolic characteristics for different GBM types derived from independent human tumors with different driver mutations (Marin-Valencia et al., 2012). Detailed metabolic remodeling observed in the GBM subtype metabolic models is discussed below.

**Table 1:** **GIMME-derived key fluxes and flux ratios for GBM subtype metabolic models, Mes, PN, and Pro**.

**Fluxes and flux ratios by GBM subtype metabolic models**	**Mes**	**PN**	**Pro**	**Experimental results for GBM**	**Healthy Brain (*iMS570*) (Sertbas et al., 2014)**
Lactate production rate (r_11_+ r_56_)	1.678	1.691	1.676	1.336 (DeBerardinis et al., 2007)	0.011
Pyruvate carboxylase flux/glucose uptake rate (r_12_)/(r_596_+ r_597_)	0	0	0	0–0.227 (Portais et al., 1993)	0.223
Oxidative PPP rate/glucose uptake rate (r_17_+ r_61_)/(r_596_+ r_597_)	0.052	0.067	0.060	0.060 (DeBerardinis et al., 2007)	0.055
Non-oxidative PPP rate (nucleotide precursor) (r_21_+ r_65_)	0.015	0.019	0.017	Increase compared to healthy brain (Wolf et al., 2010)	0.001
Oxidative metabolism (TCA) flux (r_25_+ r_69_)	0.059	0.064	0.063	Decrease compared to healthy brain (Wolf et al., 2010; Ru et al., 2013)	0.117
Acetyl-CoA flux as a lipid precursor (r_28_+ r_72_)	0.054	0.061	0.059	Increase compared to healthy brain (Wolf et al., 2010; Boroughs and DeBerardinis, 2015)	0.003
Anaplerotic reaction through glutaminolysis (r_89_+ r_90_+ r_92_+ r_93_)	0.072	0.071	0.072	0.039–0.078 (Portais et al., 1993)	–
Anaplerotic flux relative to citrate synthase (CS) activity. (r_89_+ r_90_+ r_92_+ r_93_)/(r_25_+ r_69_)	1.232	1.111	1.143	0.940–1.800 (Maher et al., 2012)	–
Acetyl-CoA carboxylase rate as the reaction initiating fatty acid synthesis (r_289_)	0.037	0.031	0.037	Increase compared to healthy brain (Wolf et al., 2010)	0.007
NH_3_ release flux (r_607_)	0.149	0.145	0.149	0.023 (DeBerardinis et al., 2007)	–
Growth rate (e_46_)	0.0069	0.0057	0.0069	0.0006–0.0095 (Perego et al., 1994; Pennington et al., 2006; Wang et al., 2009; Stensjoen et al., 2015)	–

*Rate units of metabolic reaction fluxes and growth rates are in mmol/gDW/h and 1/h respectively. In-silico flux values and ratios are compared. Corresponding reactions for reaction IDs can be found in Supplementary File 1*.

#### Aerobic glycolysis and pyruvate branch point

One of the most known metabolic alterations observed in cancer metabolism is related to aerobic glycolysis, which is also called Warburg effect. This phenomenon is characterized by a high rate of glucose consumption, which is mostly metabolized in glycolysis rather than in mitochondrial oxidative phosphorylation even in aerobic conditions, resulting in a high rate of lactate production (Warburg, 1956; Wolf et al., 2010). All *in*-*silico* GBM subtype metabolic phenotypes computed in this study exhibited the Warburg effect with a high rate of lactate production and comparatively low tricarboxylic acid (TCA) cycle activity. Flux values of the lactate production (r_11_+ r_56_) by GBM metabolic models were around 1.68 mmol/gDW/h for all GBM subtypes (Table 1).

Unlike initial cancer studies which reports that the glycolytic phenotype in cancer is due to a permanent impairment of mitochondrial oxidative phosphorylation (Zheng, 2012), both recent *in-vitro* and *in-vivo* studies demonstrate that oxidative metabolism in GBM is more active than thought (Maher et al., 2012; Marin-Valencia et al., 2012). ^13^C-labeled nutrient experiments show that glucose is metabolized through pyruvate dehydrogenase rather than pyruvate carboxylase in GBM cells. Acetyl-CoA produced from pyruvate dehydrogenase reaction then enters the TCA cycle (Maher et al., 2012; Marin-Valencia et al., 2012). Our results, in agreement with the literature, show an active flux for pyruvate dehydrogenase reaction (r_13_+ r_57_), with flux values 0.073, 0.055, and 0.073 mmol/gDW/h for Mes, PN and Pro respectively. GBM subtype metabolic models also exhibit a much lower flux in pyruvate carboxylase reaction (r_12_) (Table 1) with respect to the pyruvate dehydrogenase reaction flux, which confirms the fact that glucose metabolism does not significantly contribute to anaplerosis in GBM cells (DeBerardinis et al., 2007). Acetyl-CoA produced from pyruvate dehydrogenase and oxaloacetic acid (OAA) generate citrate via citrate synthase reaction, which is the first reaction of the TCA cycle (Figure 2). In all GBM subtype models, citrate synthase reaction (r_25_+ r_69_) is active but possesses a very low flux value compared to the healthy brain metabolic model (Table 1). Furthermore, following reactions of TCA cycle, which are conversion of citrate to isocitrate (r_26−27_+ r_70−71_) and then alpha-ketoglutarate (α-KG) (r_30−32_+ r_73−75_), carried much lower flux than the citrate synthase reaction (see Supplementary File 1 for complete flux distributions of the GBM models). One of the most known features in malignant gliomas including GBM is the mutation in isocitrate dehydrogenase (IDH) gene. While wild type IDH1 and IDH2 convert isocitrate to α-KG resulting in NADPH production, mutant IDH2 and especially IDH1 convert isocitrate to 2-hydroxyglutarate known as an oncometabolite, without producing NADPH (Dunn et al., 2012). Although the GBM metabolic models do not include mutant IDH genes and the related reaction, low flux values for conversion of isocitrate to α-KG (r_31−32_+ r_74−75_) can be explained by insufficiency in the wild type isocitrate dehydrogenase genes.

#### Glutaminolysis

Glutaminolysis is one of the key pathways in GBM since it provides glutamine as an alternative carbon source for TCA cycle (Wolf et al., 2010; Ru et al., 2013). Several experimental studies were performed to reveal the role of glutaminolysis in brain tumors (Wise et al., 2008; Yang et al., 2009; Chinnaiyan et al., 2012). Glutamine is not only the nitrogen source for nucleotide synthesis or maintenance of non-essential amino acid pools, but also the carbon and energy source which can replenish the TCA cycle intermediates in GBM cells (DeBerardinis et al., 2007; Maher et al., 2012; Ru et al., 2013). Glutamine is converted to glutamate by glutaminase reaction (r_96_+ r_97_) and then to α-KG by the anaplerotic glutamate dehydrogenase reaction (r_89_+ r_90_+ r_92_+ r_93_) in order to replenish the TCA cycle intermediates (see Table 1 for flux values).

Glutamate derived α-KG is also produced by transamination reactions (DeBerardinis et al., 2007; Yang et al., 2009) while non-essential amino acids such as aspartate and alanine are produced (see Supplementary File 1 for flux values of aspartate and alanine metabolisms). After replenishing α-KG by glutaminolysis, turnover of the TCA cycle is completed by converting α-KG to succinate, fumarate, malate and OAA respectively. All GBM subtype metabolic models exhibit active and similar flux values for the conversion of α-KG to malate (r_33−36_ and r_76−79_, see Supplementary File 1 for detailed flux values). Malate is both converted to OAA (r_37_+ r_38_+ r_80_) to complete turnover of the TCA cycle, and converted to pyruvate by malic enzyme reaction (r_39_+ r_82_), resulting in NADPH production. All GBM subtype metabolic models exhibit similar flux value (around 0.066 mmol/gDW/h) for malic enzyme reaction. In agreement with this finding, labeled glutamine experiments showed that labeled carbon was observed in lactate derived from glutamine through malic enzyme reaction (DeBerardinis et al., 2007). Ratio of the contribution of glutaminolysis and glycolysis to pyruvate pool, (r_39_ + r_82_)/(r_10_ + r_55_), was around 1/25 for GBM subtypes. Flux values for the conversion of the malate to OAA by malate dehydrogenase reaction (r_37_ + r_38_ + r_80_) were around 0.012 for GBM subtypes. As a result, major sources of the acetyl-CoA and OAA pool used in TCA cycle were respectively the pyruvate dehydrogenase from glycolysis and anaplerotic flux from glutaminolysis, which was found to be consistent with both *in-vitro* and *in-vivo* experiments (DeBerardinis et al., 2007; Yang et al., 2009; Maher et al., 2012; Marin-Valencia et al., 2012). The other source for OAA pool, pyruvate carboxylase, was found to have very low flux in our results, in accordance with the literature. Although, the turnover of the TCA cycle can be completed in GBM subtype metabolic models, ATP production fluxes from glycolysis were considerably higher than ATP production fluxes from oxidative phosphorylation pathway. ATP production fluxes from glycolysis (r_7_ + r_10_ + r_52_ + r_55_) and oxidative phosphorylation (r_45_ + r_88_) were around 2.7 and 0.4 mmol/gDW/h respectively, which shows the phenomena that although the turnover of TCA cycle can be completed, major energy source is aerobic glycolysis in GBM cells. The flux value of ATP production from oxidative phosphorylation for GBM subtypes were calculated to be 3-fold lower than the value calculated by the healthy brain metabolic model (1.236 mmol/gDW/h; Sertbas et al., 2014).

#### Precursors for tumor proliferation

In addition to energy metabolism, increased aerobic glycolysis and glutaminolysis also provide macromolecule precursors required for cell proliferation in tumors (Wolf et al., 2010; Chinnaiyan et al., 2012). Ribose-5-phosphate (R5P), produced through pentose phosphate pathway (PPP), is used as a nucleotide precursor. R5P is also used in cancer diagnosis as a tumor biomarker for its excess molecular level (Iqbal and Bamezai, 2012). Flux values of the R5P isomerase reaction (r_21_ + r_65_) producing R5P were higher for all GBM subtype metabolic models than healthy brain metabolic model (Table 1). Fatty acid synthesis relies on citrate exported from the mitochondria to cytoplasm (DeBerardinis et al., 2007; Wolf et al., 2010). The exported citrate from TCA cycle is converted to acetyl-CoA by ATP citrate lyase reaction (r_28_ + r_72_), which is the precursor for fatty acid, thereby lipid synthesis (Table 1). All GBM subtype models had a higher flux value than healthy brain metabolic model for the acetyl-CoA carboxylase reaction (r_289_), which is the first committed step for the fatty acid synthesis (Table 1). In addition to fatty acid precursors, lipid synthesis in proliferative GBM cells requires a large amount of NADPH since it is the electron donor for fatty acid synthesis (Wolf et al., 2010; Ru et al., 2013). The sources of the NADPH in the GBM models are oxidative arm of the PPP (r_17_ + r_61_), anaplerotic reaction through glutaminolysis (r_89_ + r_92_) and malic enzyme reaction (r_39_ + r_82_), which are all active for GBM subtype metabolic models (see Supplementary File 1 for flux values). A high enough NADPH supply by malic enzyme flux was reported for fatty acid synthesis, together with a glutaminolytic flux higher than PPP flux for NADPH generation (DeBerardinis et al., 2007). Our results report about 50% contribution by the glutaminolysis and 25% contribution by malic enzyme and PPP, in agreement with literature.

We found around 20% less growth rate in PN compared to the other subtypes (Table 1), which is in perfect agreement with the clinical observation that patients with PN subtype GBMs have longer survival (Lee et al., 2008; Verhaak et al., 2010). Experimental growth rates derived from doubling time (t_d_) using the formula ln2/t_d_ (Stensjoen et al., 2015) for GBM cells were also used to compare with *in-silico* derived growth rates. Growth rates of GBM subtype metabolic models are in the range obtained by both *in-vitro* and *in-vivo* experimental studies (Perego et al., 1994; Pennington et al., 2006; Wang et al., 2009; Stensjoen et al., 2015).

### Effect of platform difference and dataset difference on simulation results

**Figure 3:**
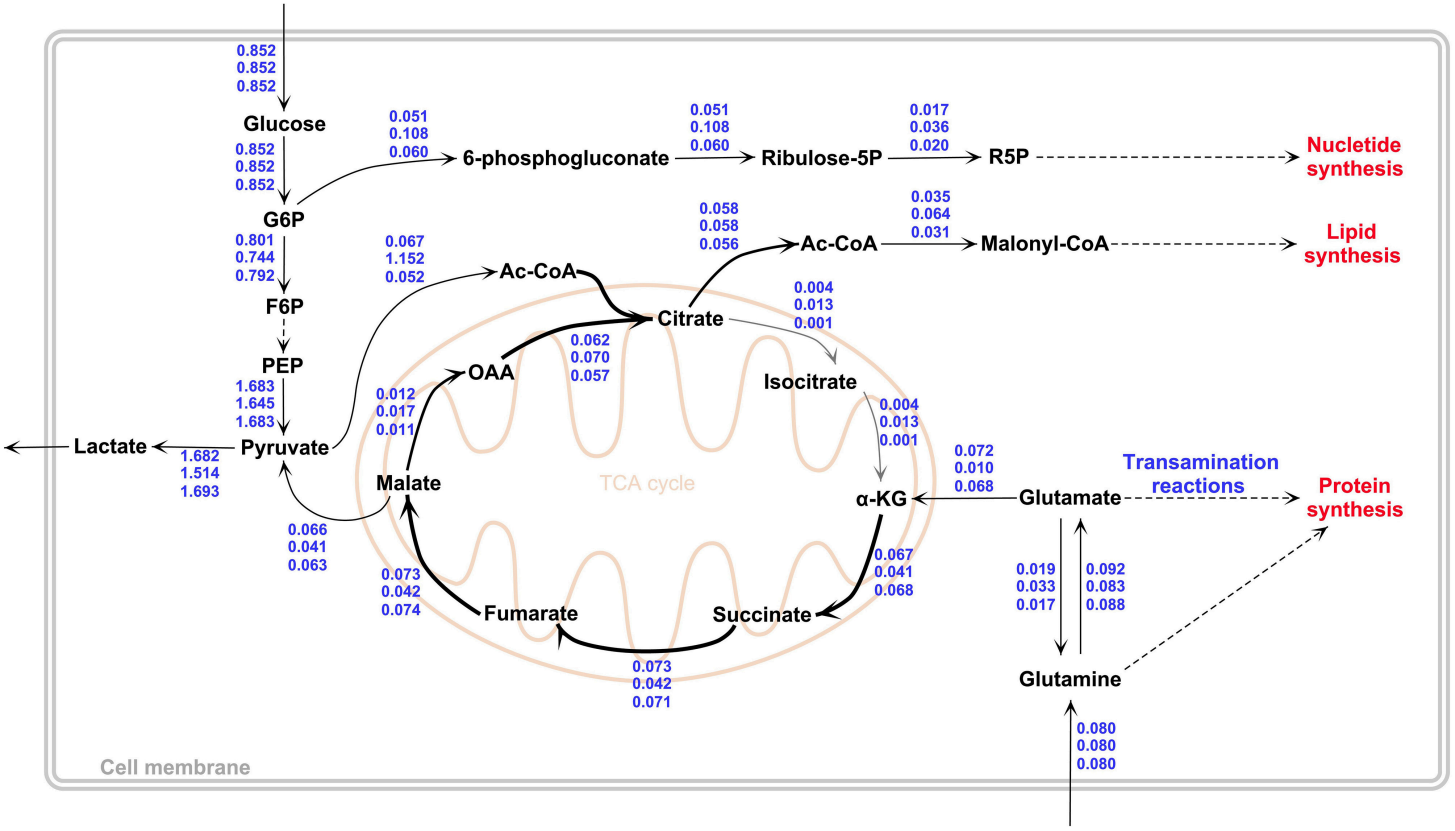
**Flux values of the *in-silico* GBM models by GIMME**. Values indicate fluxes for “the mean of the three GBM subtypes” (top, based on GSE13041-GPL96), “the metabolic model obtained using different microarray platform but from the same dataset as GBM subtypes” (middle, based on GSE13041-GPL570) and “the metabolic model obtained using the same platform as GBM subtypes but from a different dataset” (down, based on GSE13276-GPL96). Results show that constraining the model with different GBM transcriptome datasets leads to very similar flux profiles. The figure was drawn in PathVisio 3 toolbox (Kutmon et al., 2015).

In order to demonstrate the robustness of the results, transcriptome data from a different microarray platform (GPL570) but from the same dataset (GSE13041) and from the same platform (GPL96) but from a different dataset (GSE13276) were additionally used to derive GBM-specific metabolic models and calculate corresponding flux distributions (see Materials and Methods for details). This is an important issue to be considered to validate our results since platform or laboratory differences may cause serious reproducibility problems in microarray experiments (Draghici et al., 2006). No sub-type differences were accounted in these calculations. The calculated fluxes are depicted in Figure 3.

When the flux ratios reported in Table 1 were calculated for the new dataset from the same platform, a very similar profile to PN subtype results was observed, with the same *in-silico* growth rate. A classification analysis of the transcriptome data of this dataset with the PN dataset via Fisher discriminant analysis method revealed that the data of the new dataset had an acceptable degree of similarity to the PN data at transcriptome level. Based on these results, it is shown that different GBM datasets give consistent results in terms of the calculated flux phenotypes (Figure 3).

On the other hand, the use of data from the different microarray platform resulted in slightly different quantitative results, albeit not deviating from the flux rerouting behavior depicted in Figure 2. The differences include a higher growth rate (0.00118 1/h) and a higher flux to lipid metabolism through Acetyl-CoA. The lactate production rate was lower (1.51 mmol/gDW/h) then the GBM subtypes, whereas pyruvate dehydrogenase reaction and citrate synthase reaction fluxes were higher than the GBM subtypes, which were 0.152 and 0.070 mmol/gDW/h respectively (Figure 3). Furthermore, the conversion rates of citrate to α-KG (r_26−27_ + r_70−71_ and r_30−32_ + r_73−75_) and ATP production flux via oxidative phosphorylation (r_45_ + r_88_) were higher than GBM subtypes (see Supplementary File 1 for complete flux distributions of the GBM models). This shows that the metabolic model obtained using the expression data from the different platform gives TCA cycle flux more active than other *in-silico* models (Figure 3).

### Effect of transcriptome-based model-generation algorithms on simulation results

The data of GSE13276 includes also data for a reference state, which is required for MADE simulations. Therefore, this dataset was also used by MADE algorithm and compared with the results obtained by GIMME from the same dataset to enable a validation of GIMME-based flux results (see Materials and Methods). The comparison of resulting flux distributions allowed to check if there is any difference between the algorithms mapping gene expression data to the brain metabolic network, *iMS570*^*g*^. Growth rate, the objective function, was calculated to be the same (0.057 1/h) by both GIMME and MADE. Although there are some significantly different flux values for same reactions generated by GIMME and MADE, MADE-based metabolic model obeys the GBM metabolic remodeling depicted in Figure 2. For instance, citrate synthase reaction, the first reaction of the TCA cycle, is less active in MADE-based metabolic model. Citrate lyase reaction (r_28_ + r_72_) producing acetyl-CoA as the precursor for fatty acid is active for all GIMME-based metabolic models; whereas flux value of this reaction is zero for MADE. TCA cycle behavior differs quantitatively for GIMME and MADE-based models. After maximizing biomass growth rate as objective function, the minimization of Euclidean norm of internal fluxes was applied in GIMME to narrow flux ranges of reactions due to alternate optima (Cakir et al., 2007; Tarlak et al., 2014), which shows more realistic results. MADE algorithm automatically generates context-specific flux distribution and we could not apply a second optimization step to minimize the Euclidean norm of internal fluxes. This can be the reason of the significantly different flux values for same reactions generated by GIMME and MADE (for detailed flux distributions see Supplementary File 1).

## Discussion

This study provides GBM-specific genome scale metabolic models, derived from a brain-specific metabolic network. A growth reaction for tumor proliferation is implemented based on the lipid and protein content of white matter, where the GBM arises. By incorporating appropriate constraints from the literature, different GBM datasets were shown to predict similar metabolic flux reroutings, both validating our results and providing a proof of data consistency over transcriptome datasets. Moreover, the effect of different computational methods to incorporate transcriptome data with genome-scale models was investigated, and the two different methods, GIMME and MADE, which differ considerably in terms of dealing with the gene expression data, give similar qualitative results. Although there are several studies to apply genome-scale constraint-based modeling to cancer metabolism, an analysis of brain tumors with this approach is scarce. Only a recent study analyzed GBM with the FBA approach, by using a metabolic model with 147 genes and 12 pathways, without the incorporation of gene expression data (Bhowmick et al., 2015). Our work provides a much more comprehensive coverage of brain tumor metabolism. Correct predictions of flux distributions in glycolysis, glutaminolysis, TCA cycle and lipid metabolism discussed in the paper validate the reconstructed GBM specific models for further use of these models in future to simulate more specific metabolic patterns for GBM, or to predict drug targets.

When the constraints are directly applied to İ*MS570*^*g*^ without any incorporation of transcriptome data constraint, the calculated fluxes show some basic characteristics of GBM such as high glycolysis rate and increased lactate production and reduced TCA cycle activity, but missed to capture other basic GBM remodeling patterns such as the contribution of glutaminolysis to TCA cycle and a lower activity of oxidative phosphorylation. This meant higher contribution of glutamate and glutamine to growth for the purely constrained model, leading to an almost doubled growth rate compared to GBM subtype metabolic models. Therefore, some GBM patterns are observed solely because of the change in experimental constraints to GBM-specific values and change in the objective function (increased flux toward biomass precursors), but it is the use of measurement and gene expression constraints together that leads to a better prediction of GBM flux remodeling. This also shows the importance of incorporating gene expression data for flux calculations.

Another commonly used method for constraint-based genome-scale metabolic modeling is the sampling of solution space. The solution space is randomly uniformly sampled for a high number of flux vectors rather than searching the space for an optimum flux vector (Thiele et al., 2005; Megchelenbrink et al., 2014). The approach is especially preferred for the analysis of mammalian metabolism. Here, we re-analyzed all GIMME-derived GBM-specific metabolic models with the sampling approach by constraining the growth rate between the optimum value and 80% of the optimum. Resulting flux values and flux reroutings were similar to the values reported in Table 1, Figure 2 (results not shown). Interestingly, the flux values obtained for the effect of a different microarray platform (GPL570) were more similar to the values obtained for the GBM subtypes (Figure 3). For example, a lower flux pentose phosphate pathway was obtained for this platform with sampling approach.

## Author contributions

TÇ conceived and designed the study, EO performed all simulations, TÇ, EO analyzed the results and wrote the manuscript.

### Conflict of interest statement

The authors declare that the research was conducted in the absence of any commercial or financial relationships that could be construed as a potential conflict of interest.
